# A 90 day chronic toxicity study of Nigerian herbal preparation DAS-77 in rats

**DOI:** 10.1186/1472-6882-12-79

**Published:** 2012-06-28

**Authors:** Saheed O Afolabi, Abidemi J Akindele, Olufunsho Awodele, Chidozie C Anunobi, Olufunmilayo O Adeyemi

**Affiliations:** 1 Department of Pharmacology, Faculty of Basic Medical Sciences, College of Medicine, University of Lagos, Lagos, P. M. B, 12003, Nigeria; 2 Department of Morbid Anatomy, Faculty of Basic Medical Sciences, College of Medicine, University of Lagos, Lagos, P. M. B, 12003, Nigeria

**Keywords:** DAS-77, Herbal preparation, Toxicological assessment, Acute toxicity, Chronic toxicity

## Abstract

**Background:**

The herbal preparation DAS-77, used for the treatment of various ailments in Nigeria, contains the milled bark of *Mangifera indica* L. and root of *Carica papaya* L. Toxicological assessment of the preparation was carried out in this study.

**Methods:**

In the acute toxicity study, DAS-77 was administered to mice *p.o.* up to 20 g/kg in divided doses and *i.p.* at 250–3000 mg/kg. Mortality within 24 h was recorded. In the chronic toxicity study, rats were treated *p.o.* for 90 days at doses of 80, 400 (therapeutic dose, TD) and 2000 mg/kg. By 90 days, animals were sacrificed and blood samples collected for hematological and biochemical analysis. Organs were harvested for weight determination, antioxidants and histopathological assessments.

**Results:**

DAS-77 did not produce any lethality administered *p.o.* up to 20 g/kg in divided doses but the *i.p.* LD_50_ was 1122.0 mg/kg. At TD, DAS-77 produced significant (*p* < 0.05) reductions in body weight, food intake and K^+^, and increases in ovary weight, neutrophils and HDL, which were reversible. Histopathological presentations were generally normal. Effects at the other doses were comparable to those at TD except for reversible increases in antioxidants in the liver, kidney and testes, and sperm abnormality, and reductions in liver enzymes, sperm motility and count.

**Conclusions:**

Findings in this study revealed that DAS-77 is relatively safe with the potential for enhancing *in vivo* antioxidant activity. However, possibly reversible side-effects include electrolyte imbalance and sterility in males.

## Background

Herbal medicines as preparations derived from naturally occurring plants with medicinal or preventive properties are a major component in all indigenous peoples’ traditional medicine, including Ayurvedic, homeopathic, naturopathic, traditional oriental, Native American [1] and African medicine. Aside from the discovery of numerous orthodox drugs from the study of traditional cures and folk knowledge, the efficacy of a number of botanicals has been proven scientifically. Based on a number of factors like cultural significance, accessibility, affordability and perceived safety, herbal medicines remain relevant in the primary health care of indigenous populations worldwide. The World Health Organization (WHO) estimates that 4 billion people, about 80% of the world’s population, use herbal medicines for some aspect of primary health care [1]. It is estimated that 70% of indigenous populations in developing countries rely on phytotherapy [2] and 25% of drugs in typical western pharmacies are plant-derived [3]. In view of their availability for sale at a wide range of retail outlets, the extent of their advertisement in the popular media, and the entrance of several major pharmaceutical companies into the business of producing phytomedicinal products, phytomedicines have clearly re-emerged into the mainstream as a point of call in primary health care [4-6].

Considering the challenges confronting the appropriate delivery of official health care to millions of people in remote and rural communities which serve as abode for over 70% of the population, including socio-economic demands for adequate pharmaceutical supplies, prevalent transportation difficulties, shortage of needed expertise for rational use of drugs, shortage and cost of orthodox products [2], the most viable way to bridge the gap in medicare is herbal medicines. The realities on ground pose challenges to scientists and governments of developing countries with respect to the development and production of standardized herbal medicines. WHO has advocated for the proper identification, sensible exploitation, scientific development and appropriate utilization of herbal medicines which provide safe and effective remedies in medicare [2]. Herbal remedies are generally regarded as safe and are promoted to the public as being “natural” and completely “safe” [7], owing to long history of use [8]. The surge in popularity and patronage of herbal medicines necessitate concern based on adverse effects of potentially toxic constituents in plants (e.g. aristolochic acids, pyrrolizidine alkaloids, benzophenanthrine alkaloids, lectins, viscotoxins, saponins, diterpenes, cyanogenetic glycosides and furonocoumarins) which can be fatal [9]. Pharmacological and toxicological evaluations of medicinal plants are essential for drug development [10-12]. So much has been done in screening herbal medicines for efficacy based on traditional claims while less emphasis is placed on the issue of safety, as reports of efficacy far outnumber those of toxicity. For example, of 112 abstracts accepted for presentation at the 2011 conference of the West African Society for Pharmacology (WASP) held at the Kwame Nkrumah University of Science and Technology (KNUST), Kumasi, Ghana, 70 (62.5%) were on herbal medicines while 42 (37.5%) were on other aspects of Pharmacology. Of the 70 on herbal medicines, 56 (80%) attested to efficacy while 14 (20%) raised issues of safety with 8 being on both efficacy and safety. The summary is that presentations attesting to beneficial effects of herbal remedies were four times those cautioning about their safety (*personal communication by the President of WASP, Prof. Helen Kwanashie*).

DAS-77 is a herbal preparation that contains the milled dried callous bark of mango (*Mangifera indica* Linn., Anacardiaceae) and the dried root of pawpaw (*Carica papaya* Linn., Caricaceae). It is used to treat diverse ailments in Nigeria including ulcers, diarrhea, sexually transmitted infections, dysmenorrhea, cholera, stomach disorders, dysentery and hemorrhoids. Due to multiplicity of usage and tendency for prolonged use, the herbal preparation was investigated in this study for possible chronic toxicity effects using a 90 day administration schedule.

## Methods

### Herbal product

The herbal preparation DAS-77 is a product of Doynik Ventures, Ijoko-Lemode, via Sango Ota, Ogun State, Nigeria. It contains the milled young callous bark of mango (*Mangifera indica*) and the dried root extract of pawpaw (*Carica papaya*) (1:1). The product is a light brown, coarse powder with a pungent smell, and solution pH 8.5. DAS-77 was constituted in distilled water before administration to experimental animals.

### Experimental animals

Inbred Wistar rats and mice of both sexes, averagely weighing 200 g and 15 g respectively, used in this study were obtained from the Laboratory Animal Center of the College of Medicine, University of Lagos, Lagos, Nigeria. The animals were maintained under standard environmental conditions (23-25°C, 12 h/12 h light/dark cycle) and had free access to standard rodent pellet diet (Livestock Feeds PLC, Ikeja, Lagos State, Nigeria) and water *ad libitum*. The protocol used in this study was approved by the Experimentation Ethics Committee on Animal Use of the College of Medicine, University of Lagos, Lagos, Nigeria, and it was in accordance with the United States National Institutes of Health Guidelines for Care and Use of Laboratory Animals in Biomedical Research [13].

### Phytochemical screening

Preliminary phytochemical screening (qualitative and quantitative) of DAS-77 was done according to the methods of Harborne [14], Trease and Evans [15] and Edeoga *et al*. [16].

### Acute toxicity study

Mice were generally fasted for 12 h prior to the commencement of this test. A group of animals received DAS-77 orally (*p.o.*) at a dose of 20 g/kg in divided doses. Six other groups of mice (n = 5) separately received distilled water 10 ml/kg and DAS-77 at doses of 250, 500, 1000, 2000, and 3000 mg/kg intraperitoneally (*i.p.*). Mice were generally observed for toxic symptoms and behavioural changes (sedation, hyperactivity, diarrhea, writhing, piloerection, restlessness *etc.*) for 2 h post-administration. The median lethal dose (LD_50_) was estimated by the log dose-probit analysis method [17,18] based on mortality recorded within 24 h.

### Chronic toxicity study

A total of 80 rats were randomly allotted to 4 groups of 14 male and 6 female animals each housed separately in polypropylene cages. The animals were daily treated *p.o.* with distilled water (control) and DAS-77 at doses of 80, 400, and 2000 mg/kg (representing one-fifth of the pharmacologically active dose, the pharmacologically active dose, and five times the pharmacologically active dose respectively [19,20] for 90 days. The pharmacologically active dose was the most effective dose in the investigation of the analgesic and antiulcer activities of DAS-77 in our laboratory (unpublished data).

Rats were weighed weekly and observed for behavioral changes, feeding and drinking habits, and general morphological changes. At the end of the 90 day treatment period, 10 rats from each group were anaesthetized by *i.p.* administration of 5 ml/kg of a solution of 1% chloralose in 25% urethane (w/v) [8]. Blood samples were collected from rats by cardiac puncture into EDTA (ethylenediamine-tetra acetate) sample bottles for hematological analysis and into plain sample bottles for serum generation for biochemical analysis. Serum was obtained after allowing blood to coagulate for 30 min. and centrifugation. Semen was obtained from male rats for assessment of sperm motility, count and morphology according to the method of Cheesbrough [21]. After sacrificing the experimental animals, vital organs including the heart, lungs, spleen, kidneys, lungs, testicles, ovaries, epididymis and pancreas were harvested, carefully examined for gross lesions, and weighed. The weight of each organ was standardized to 100 g body weight of each animal. Samples were taken from each of the organs for determination of *in vivo* antioxidants and malondialdehyde (MDA). The remnants of the organs were preserved in 10% formol-saline for histopathological assessment.

Mortality in each treatment group was recorded during the course of the 90 day administration of DAS-77 and reversibility period of 30 days in which animals were not treated with the herbal preparation. At the end of the reversibility period, rats were sacrificed and assays conducted in the main study were done.

### Hematological analysis

Blood samples were analyzed using established procedures and automated hematology analyzer. Parameters evaluated include packed cell volume (PCV), red blood cell (RBC) count, hemoglobin (Hb), platelet count, total and differential white blood cell (WBC) count, mean cell hemoglobin concentration (MCHC), mean red cell volume (MCV), and mean cell hemoglobin (MCH).

### Biochemical analysis

Serum samples were analyzed for creatinine, urea, albumin, total protein, cholesterol, high density lipoprotein (HDL), low density lipoprotein (LDL), triglycerides (TG), glucose, uric acid, alkaline phosphatase (ALP), aspartate transaminase (AST), alanine transaminase (ALT), total bilirubin, and direct bilirubin using Roche and Cobas commercial kits and Roche/Hitachi 904 automated analyzer. Serum electrolytes concentration was determined by established methods; sodium and potassium concentration by flame photometry, chloride and bicarbonate concentration by titrimetric method, and calcium concentration by cresol phthalein complexone method [22-24].

### Sperm analysis

Sperm analysis to assess seminal fluid for motility, count and morphology was carried out according to the methods of Cheesbrough [21] and Ogli et al. [25]. Male rats were sacrificed and strapped astride on their back on dissecting board. The testis was removed with its ipsilateral epididymis into a beaker after incision on the right scrotum. Subsequently, semen was expelled out of the epididymis into a beaker placed in water bath at 36°C [25].

### Sperm motility

Semen (10–15 μl) was placed on a slide in a way that the spermatozoa were evenly distributed and covered with a glass. After appropriate focus, several fields of the specimen were assessed for motility using the 40 × objective of the microscope. The number of motile cells was noted out of a total of 100 spermatozoa.

### Sperm count

Using sodium bicarbonate-formalin diluting fluid, a 1 in 20 dilution of semen was carried out with thorough mixing. An improved Neubauer ruled chamber was filled with well-mixed diluted semen using a Pasteur pipette. The number of spermatozoa in an area of 2 sq mm was counted using the 10 × objective of the microscope after 3–5 min. Estimation of the number of spermatozoa in 1 ml of fluid was done by multiplication of the number counted by 100 000.

### Sperm morphology

A thin smear of the liquefied well-mixed semen, made on a slide, was fixed with 95% v/v ethanol while still wet for 5–10 min. This was allowed to air-dry after which it was washed with sodium bicarbonate-formalin solution. This was to remove any present mucus. Rinsing with water was carried out many times. Subsequently, the smear was covered with dilute carbon fuchsin (1 in 20). This was allowed to stain for 3 min and the stain was washed off with water. Dilute Loeffler’s methylene blue (1 in 20) was used to cover the smear for 2 min to achieve counterstaining. This was washed off with water, drained and allowed to dry. Normal and abnormal spermatozoa were examined using the 40 × objective of the microscope. Estimation of percentage normal and abnormal morphology was done from the counting of hundred spermatozoa.

### Measurement of in vivo antioxidants and malondialdehyde (MDA) levels

Reduced glutathione (GSH), superoxide dismutase (SOD), catalase (CAT), glutathione peroxidase (GPx), and MDA were determined according to established methods [26-29]. The Biuret method was used for the determination of total protein [30].

### Histopathological assessment

The various tissues obtained from experimental animals fixed in 10% formol-saline were dehydrated in graded alcohol, embedded in paraffin, and cut into 4–5 μm thick sections. Hematoxylin-eosin was used to stain the sections for photomicroscopic assessment using a Model N-400ME photomicroscope (CEL-TECH Diagnostics, Hamburg, Germany) [31,32]. Slides were examined using the × 40, × 100, and × 400 objectives.

### Statistical analysis

Results are expressed as mean ± SEM. Data analysis was carried out using One-way ANOVA followed by Dunnet’s and Bonferroni posttests using GraphPad Prism 5 (GraphPad Software Inc., CA, USA). Significance was considered at values of *p* < 0.05.

## Results

### Phytochemical screening

Preliminary phytochemical screening of DAS-77 showed the presence of tannins (3.26%), saponins (2.32%), phenols (1.31%), flavonoids (0.54%) and alkaloids (0.04%). Percentages represent the crude yield of the phytoconstituents.

### Acute toxicity

No mortality and visible signs of toxicity were observed upon administration of DAS-77 *p.o.* up to 20 g/kg in divided doses. Administered *i.p.*, mortality was 0% at the lowest dose of 250 mg/kg and 100% at the highest dose of 3000 mg/kg, with the LD_50_ graphically estimated to be 1122.0 mg/kg. Writhing, grooming, increased locomotor activity, and convulsion were the behavioral manifestations observed with the *i.p.* route.

### Effect of DAS-77 on body weight, food and water intake

**Figure 1:**
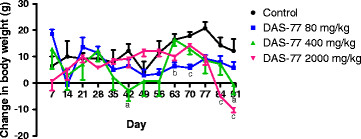
**Effect of DAS-77 on body weight of male rats. Values are presented as mean ± SEM (n = 7).**^a^*p* < 0.05, ^b^*p* < 0.01, ^c^*p* < 0.001 vs. control (One-way ANOVA followed by Dunnet’s posttest).

In respect of male rats, compared to control, DAS-77 at the dose of 80 mg/kg caused significant reduction (*p* < 0.01, 0.001) in change in body weight on days 63 and 70. The same trend of effect was obtained with DAS-77 at doses of 400 mg/kg (*p* < 0.05) and 2000 mg/kg (*p* < 0.001) but on days 42 and 90, and 84 and 90 respectively (Figure 1).

**Figure 2:**
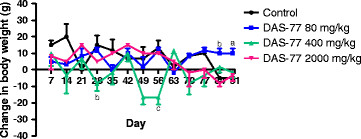
**Effect of DAS-77 on body weight of female rats.** Values are presented as mean ± SEM (n = 3). ^a^*p* < 0.05, ^b^*p* < 0.01, ^c^*p* < 0.001 vs. control (One-way ANOVA followed by Dunnet’s posttest).

Concerning female rats, compared to control, DAS-77 at the dose of 80 mg/kg caused significant increase (*p* < 0.05, 0.01) in change in body weight on days 84 and 90. At the dose of 400 mg/kg, the herbal remedy elicited significant reduction (*p* < 0.01, 0.001) on days 28 and 56. DAS-77 at the dose of 2000 mg/kg did not produce any significant effect on change in body weight all through the treatment period (Figure 2).

**Figure 3:**
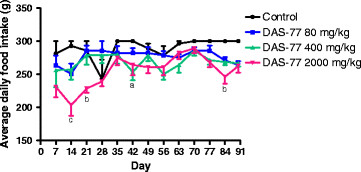
**Effect of DAS-77 on daily food intake.** Values are presented as mean ± SEM (n = 7). ^a^*p*< 0.05, ^b^*p* < 0.01, ^c^*p* < 0.001 vs. control (One-way ANOVA followed by Bonferroni posttests).

In respect of food intake, DAS-77 produced significant effect (*p* < 0.05, 0.01, 0.001) on average daily food intake at the dose of 2000 mg/kg only. At this dose, there were reductions in average daily food intake on days 14, 21, 42 and 84 compared to control (Figure 3).

**Figure 4:**
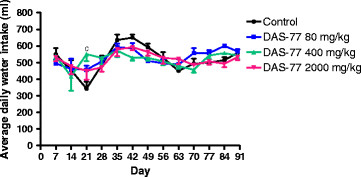
**Effect of DAS-77 on daily water intake.** Values are presented as mean ± SEM (n = 7). ^c^*p* < 0.001 vs. control (One-way ANOVA followed by Bonferroni posttests).

Concerning water intake, DAS-77 at the dose of 400 mg/kg caused significant increase (*p* < 0.001) in average daily water intake on day 21 compared to control. The herbal remedy at doses of 80 and 2000 mg/kg did not elicit any significant effect all through the treatment period (Figure 4).

In general, considering the 90 day end-point, DAS-77 at the dose of 400 mg/kg produced a significant reduction (*p* < 0.01) in weight change (3.85 ± 1.61 g) compared to control (10.46 ± 0.97 g) while effects were not significant at 80 and 2000 mg/kg. The herbal preparation also elicited a significant reduction (*p* < 0.01, 0.001) in food intake at doses of 400 and 2000 mg/kg (268.40 ± 3.21 and 254.60 ± 6.64 g, respectively) relative to control (289.80 ± 4.46 g). DAS-77 did not produce any significant effect on water intake (Table 1).

**Table 1:** Effect of DAS-77 on animal weight, food and water intake

**Treatment**	**Dose (mg/kg)**	**Weight change (g)**	**Food intake (g)**	**Water intake (ml)**
**Distilled water**	(10 ml/kg)	10.46 ± 0.97	289.80 ± 4.46	519.10 ± 22.73
**DAS-77**	80	7.23 ± 1.13	276.40 ± 3.03	529.20 ± 13.91
**DAS-77**	400	3.85 ± 1.61^**^	268.40 ± 3.21^**^	519.00 ± 12.13
**DAS-77**	2000	5.85 ± 1.74	254.60 ± 6.64^***^	517.70 ± 11.86

Values are mean ± S.E.M (n = 10). ^**^p < 0.01, ^***^p < 0.001 vs. distilled water (One-way ANOVA followed by Dunnett’s posttest).

### Effect of DAS-77 on sperm motility, sperm count, and morphology (% abnormality)

At doses of 80 and 400 mg/kg, DAS-77 did not produce any significant effect on sperm motility, count, and abnormality. However at the dose of 2000 mg/kg, the herbal preparation significantly reduced (*p* < 0.05, 0.01) sperm motility (42.71 ± 10.33% vs. control value of 85.00 ± 6.00%) and sperm count (14.71 ± 3.00 million/ml vs. control value of 31.14 ± 2.50 million/ml), and increased sperm abnormality (12.43 ± 2.26% vs. control value of 5.14 ± 1.10%). These effects were reversed (Table 2).

**Table 2:** Effect of DAS-77 on sperm motility, count and morphology (% abnormality)

		**Main study**			**Reversibility study**		
**Treatment**	**Dose (mg/kg)**	**Sperm motility (%)**	**Sperm count (million/ml)**	**Abnormality (%)**	**Sperm motility (%)**	**Sperm counted (million/ml)**	**Abnormality (%)**
**Distilled water**	(10 ml/kg)	85.00 ± 6.00	31.14 ± 2.50	5.14 ± 1.10	81.60 ± 7.17	25.80 ± 4.28	7.20 ± 1.72
**DAS-77**	80	73.43 ± 12.61	25.57 ± 4.07	8.86 ± 2.42	72.33 ± 9.30	24.67 ± 3.53	8.33 ± 2.01
**DAS-77**	400	87.29 ± 12.05	33.00 ± 3.52	4.14 ± 1.44	70.80 ± 9.07	23.00 ± 2.54	7.40 ± 1.50
**DAS-77**	2000	42.71 ± 10.33^*^	14.71 ± 3.00^**^	12.43 ± 2.26^*^	74.25 ± 13.56	25.50 ± 5.52	5.50 ± 1.71

Values are mean ± S.E.M (n = 7, main study; n = 5, reversibility study). ^*^p < 0.05, ^**^p < 0.01 vs. distilled water (One-way ANOVA followed by Dunnett’s posttest).

### Effect on weight of vital organs (per 100 g body weight)

DAS-77 did not produce any significant effect on the weight of vital organs except in the case of the liver in which there was a significant reduction (*p* < 0.05) in weight (2.41 ± 0.10 g vs. 2.84 ± 0.05 g for control) at the dose of 2000 mg/kg and the ovaries in which there were significant increases (*p* < 0.01) in weight (0.14 ± 0.02 g and 0.14 ± 0.01 g respectively vs. 0.08 ± 0.00 g for control) at doses of 400 and 2000 mg/kg (Table 3). The effects produced in the liver and ovaries were reversed upon cessation of treatment for 30 days (Table 4). DAS-77 did not produce any significant effect on the weight of the lungs in the main study but at the dose of 400 mg/kg a significant increase (*p* < 0.05) was observed in the reversibility study (0.88 ± 0.03 g vs. 0.73 ± 0.06 g for control).

**Table 3:** Effect of DAS-77 on weight of vital organs (per 100 g body weight) in the main study

**Treatment**	**Dose (mg/kg)**	**Main Study**								
		**Liver**	**Kidney**	**Lungs**	**Heart**	**Testes**	**Spleen**	**Pancreas**	**Brain**	**Ovaries**
**Distilled water**	(10 ml/kg)	2.84 ± 0.05	0.57 ± 0.02	0.77 ± 0.04	0.37 ± 0.03	1.52 ± 0.08	0.37 ± 0.03	0.28 ± 0.02	0.63 ± 0.03	0.08 ± 0.00
**DAS-77**	80	2.80 ± 0.19	0.64 ± 0.04	0.78 ± 0.05	0.34 ± 0.02	1.78 ± 0.29	0.32 ± 0.04	0.34 ± 0.03	0.70 ± 0.02	0.10 ± 0.00
**DAS-77**	400	2.66 ± 0.09	0.71 ± 0.03	0.88 ± 0.04	0.33 ± 0.02	1.99 ± 0.08	0.31 ± 0.02	0.42 ± 0.02	0.74 ± 0.08	0.14 ± 0.02^**^
**DAS-77**	2000	2.41 ± 0.1^*^	0.65 ± 0.06	0.78 ± 0.07	0.35 ± 0.02	1.91 ± 0.13	0.30 ± 0.02	0.32 ± 0.03	0.73 ± 0.01	0.14 ± 0.01^*^

Values are mean ± S.E.M (n = 10). ^*^p < 0.05, ^**^p < 0.01 vs. distilled water (One-way ANOVA followed by Dunnett’s posttest).

**Table 4:** Effect of DAS-77 on weight of vital organs (per 100 g body weight) in the reversibility study

**Treatment**	**Dose (mg/kg)**	**Reversibility study**								
		**Liver**	**Kidney**	**Lungs**	**Heart**	**Testes**	**Spleen**	**Pancreas**	**Brain**	**Ovaries**
**Distilled water**	(10 ml/kg)	2.60 ± 0.11	0.57 ± 0.01	0.73 ± 0.06	0.36 ± 0.02	1.39 ± 0.13	0.29 ± 0.01	0.25 ± 0.03	0.78 ± 0.03	0.12 ± 0.00
**DAS-77**	80	2.53 ± 0.12	0.55 ± 0.03	0.68 ± 0.03	0.35 ± 0.02	1.21 ± 0.05	0.25 ± 0.01	0.24 ± 0.02	0.82 ± 0.04	0.10 ± 0.01
**DAS-77**	400	2.74 ± 0.12	0.62 ± 0.01	0.88 ± 0.0^*^	0.42 ± 0.03	0.95 ± 0.27	0.25 ± 0.01	0.26 ± 0.02	0.71 ± 0.04	0.12 ± 0.01
**DAS-77**	2000	2.79 ± 0.10	0.63 ± 0.05	0.83 ± 0.02	0.38 ± 0.03	1.35 ± 0.28	0.27 ± 0.02	0.34 ± 0.02	0.82 ± 0.04	0.14 ± 0.01

Values are mean ± S.E.M (n = 10). ^*^p < 0.05 vs. distilled water (One-way ANOVA followed by Dunnett’s posttest).

### Effect of DAS-77 on hematological parameters

DAS-77 did not produce any significant effect on hematological parameters after the 90 day administration except in respect of WBC count in which there was a significant decrease (*p* < 0.05) in the group treated with the herbal preparation at the dose of 2000 mg/kg (5.20 ± 0.58 × 10³/μl) when compared with the group treated with distilled water (9.40 ± 0.98 × 10³/μl) (Table 5). There was also a significant increase (*p* < 0.05, 0.01) in the proportion of neutrophils in the groups treated with DAS-77 at doses of 80 mg/kg (46.10 ± 2.16%), 400 mg/kg (56.30 ± 1.54%) and 2000 mg/kg (60.90 ± 1.38%) compared to control (39.80 ± 1.95%). The effects of DAS-77 on WBC count and proportion of neutrophils were reversed after 30 days of cessation of administration (Table 6).

**Table 5:** Effect of DAS-77 on hematological parameters in rats in the main study

**Treatment**	**Dose (mg/kg)**	**Main study**										
		**PCV (%)**	**WBC (10³/μl)**	**N (%)**	**L (%)**	**E (%)**	**RBC (10**^**6**^**/μl)**	**PLT (10**^**4**^**/μl)**	**Hb (g/dl)**	**MCV (fl)**	**MCH (pg)**	**MCHC (g/dl)**
**Distilled water**	(10 ml/kg)	42.90 ± 1.49	9.40 ± 0.98	39.80 ± 1.95	59.60 ± 2.04	1.50 ± 0.29	5.24 ± 0.35	34.15 ± 2.43	13.91 ± 0.47	83.30 ± 2.68	27.10 ± 0.89	32.42 ± 0.13
**DAS-77**	80	45.30 ± 1.37	8.84 ± 0.76	46.10 ± 2.16^*^	52.40 ± 1.93	2.75 ± 1.11	6.26 ± 0.50	31.90 ± 3.07	14.73 ± 0.44	75.00 ± 4.56	24.03 ±1.66	32.54 ± 0.05
**DAS-77**	400	42.90 ± 1.55	5.92 ± 1.60	56.30 ± 1.54^**^	56.30 ± 1.54	1.67 ± 0.67	4.57 ± 0.32	34.80 ± 1.92	13.93 ± 0.48	84.40 ±2.08	27.38 ± 0.72	32.50 ± 0.06
**DAS-77**	2000	40.60 ± 0.88	5.20 ± 0.58^*^	60.90 ± 1.38^**^	60.90 ± 1.38	3.50 ± 0.50	4.67 ± 0.12	36.04 ± 1.47	12.23 ± 1.00	87.20 ± 1.23	28.39 ± 0.41	32.60 ± 0.03

Values are mean ± S.E.M. (n = 10). ^*^p < 0.05, ^**^p < 0.01 vs. distilled water (One-way ANOVA followed by Dunnett’s posttest).

**Table 6:** Effect of DAS-77 on hematological parameters in rats in the reversibility study

**Treatment**	**Dose (mg/kg)**	**Reversibility study**										
		**PCV (%)**	**WBC (10³/μl)**	**N (%)**	**L (%)**	**E (%)**	**RBC (10**^**6**^**/μl)**	**PLT (10**^**4**^**/μl)**	**Hb (g/dl)**	**MCV (fl)**	**MCH (pg)**	**MCHC (g/dl)**
**Distilled water**	(10 ml/kg)	45.13 ± 1.16	6.91 ± 0.62	47.75 ± 3.06	51.88 ± 3.19	1.50 ± 0.05	6.93 ± 0.37	2.69 ± 0.22	14.73 ± 0.37	66.00 ± 2.30	21.53 ± 0.77	32.66 ± 0.03
**DAS-77**	80	43.78 ± 1.01	5.18 ± 0.78	45.00 ± 1.88	53.44 ± 2.04	2.25 ± 0.95	6.59 ± 0.27	3.74 ± 0.13	14.28 ± 0.32	66.78 ± 1.53	21.83 ± 0.52	32.61 ± 0.07
**DAS-77**	400	43.13 ± 1.17	6.39 ± 0.67	48.75 ± 2.26	50.13 ± 2.25	2.25 ± 0.48	6.28 ± 0.44	3.08 ± 0.14	14.10 ± 0.39	70.63 ± 3.99	23.08 ± 1.30	32.17 ± 0.04
**DAS-77**	2000	45.50 ± 0.96	5.12 ± 0.49	50.00 ± 2.79	49.83 ± 2.81	1.00 ± 0.00	6.92 ± 0.24	3.93 ± 0.24	14.83 ± 0.31	66.17 ± 1.01	21.48 ± 0.35	32.60 ± 0.04

Values are mean ± S.E.M. (n = 8). *p* > 0.05 vs. distilled water (One-way ANOVA followed by Dunnett’s posttest).

### Effect of DAS-77 on serum biochemical parameters

There was a significant increase (*p* < 0.05) in the concentration of albumin in the group treated with the herbal preparation at the dose of 80 mg/kg (37.30 ± 0.78 g/L) when compared with the group treated with distilled water (33.20 ± 1.03 g/L) (Table 7). There was also a significant increase (*p* < 0.05) in the concentration of HDL in the groups treated with DAS-77 at doses of 80 mg/kg (1.15 ± 0.05 mmol/L) and 400 mg/kg (1.11 ± 0.10 mmol/L) when compared with the group treated with distilled water (0.87 ± 0.05 mmol/L). The concentrations of AST and ALT were significantly reduced (*p* < 0.001) in the groups treated with the formulation at the dose of 2000 mg/kg (74.50 ± 8.61 u/L and 18.00 ± 2.12 u/L respectively) when compared with the group treated with distilled water (143.70 ± 11.34 u/L and 36.20 ± 4.56 u/L respectively). The effects on albumin, HDL, AST, and ALT were reversed after 30 days of cessation of administration of DAS-77 (Table 8).

**Table 7:** Effect of DAS-77 on serum biochemical parameters in rats in the main study

**Treatment**	**Dose (mg/kg)**	**Main study**														
		**Creatinine (μmol/L)**	**Urea (mmol/L)**	**Albumin (g/L)**	**Protein (g/L)**	**TC (mmol/L)**	**HDL (mmol/L)**	**LDL (mmol/L)**	**TG (mmol/L)**	**Glucose (mmol/L)**	**Uric acid (μmol/L)**	**ALP (u/L)**	**AST (u/L)**	**ALT (u/L)**	**TB (μmol/L)**	**DB (μmol/L)**
**Distilled water**	(10 ml/kg)	63.40 ± 1.06	8.69 ± 0.29	33.20 ± 1.03	72.50 ± 1.85	1.80 ± 0.16	0.87 ± 0.05	0.65 ± 0.18	0.61 ± 0.06	1.64 ± 0.29	104.20 ± 23.85	40.40 ± 4.89	143.70 ± 11.34	36.20 ± 4.56	5.70 ± 0.26	1.40 ± 0.31
**DAS-77**	80	54.80 ± 3.46	9.51 ± 0.40	37.30 ± 0.78^*^	75.80 ± 0.98	1.91 ± 0.11	1.15 ± 0.05^*^	0.59 ± 0.13	0.41 ± 0.05	1.60 ± 0.16	75.33 ± 9.17	33.07 ± 3.47	111.20 ± 14.8	31.80 ± 2.05	5.40 ± 0.22	1.10 ± 0.1
**DAS-77**	400	55.50 ± 3.21	9.93 ± 0.62	35.20 ± 1.59	74.10 ± 3.26	1.72 ± 0.15	1.11 ± 0.10^*^	0.43 ± 0.08	0.41 ± 0.07	1.58 ± 0.23	60.18 ± 5.66	34.02 ± 3.15	80.0 ± 6.21	18.2 ± 1.26	5.80 ± 0.25	1.40 ± 0.31
**DAS-77**	2000	57.00 ± 2.08	9.12 ± 0.59	35.20 ± 1.03	78.80 ± 1.71	1.77 ± 0.16	1.01 ± 0.06	0.62 ± 0.16	0.48 ± 0.08	1.57 ± 0.15	58.77 ± 4.33	40.54 ± 4.78	74.50 ± 8.61^***^	18.00 ± 2.12^***^	5.40 ± 0.22	1.30 ± 0.15

Values are mean ± S.E.M. (n = 10). ^*^p < 0.05, ^***^p < 0.001 vs. distilled water (One-way ANOVA followed by Dunnett’s posttest).

**Table 8:** Effect of DAS-77 on serum biochemical parameters in rats in the reversibility study

**Treatment**	**Dose(mg/kg)**	**Reversibility study**														
		**Creatinine (μmol/L)**	**Urea (mmol/L)**	**Albumin (g/L)**	**Protein (g/L)**	**TC (mmol/L)**	**HDL (mmol/L)**	**LDL (mmol/L)**	**TG (mmol/L)**	**Glucose (mmol/L)**	**Uric acid (μmol/L)**	**ALP (u/L)**	**AST (u/L)**	**ALT (u/L)**	**TB (μmol/L)**	**DB (μmol/L)**
**Distilled water**	(10 ml/kg)	58.75 ± 2.95	7.68 ± 0.38	39.5 ± 1.68	83.13 ± 3.14	2.4 ± 0.25	1.59 ± 0.15	0.50 ± 0.18	0.65 ± 0.10	1.93 ± 0.36	93.74 ± 8.50	28.23 ± 5.48	65.88 ± 7.87	31.75 ± 3.08	5.75 ± 0.31	1.50 ± 0.27
**DAS-77**	80	60.11 ± 2.67	7.72 ± 0.36	40.89 ± 1.35	84.11 ± 3.25	2.18 ± 0.15	1.61 ± 0.15	0.24 ± 0.04	0.70 ± 0.08	2.36 ± 0.34	85.33 ± 5.54	20.11 ± 2.57	58.56 ± 5.26	26.67 ± 1.62	5.22 ± 0.15	1.44 ± 0.18
**DAS-77**	400	54.50 ± 1.05	7.41 ± 0.52	39.00 ± 1.60	85.38 ± 3.69	2.25 ± 0.33	1.61 ± 0.32	0.70 ± 0.16	0.68 ± 0.13	1.84 ± 0.28	97.75 ± 10.75	25.69 ± 3.09	51.50 ± 4.05	33.63 ± 2.06	5.38 ± 0.26	1.50 ± 0.38
**DAS-77**	2000	55.50 ± 2.05	7.12 ± 0.31	38.50 ± 1.34	84.67 ± 6.04	1.90 ± 0.24	1.10 ± 0.18	0.47 ± 0.09	0.70 ± 0.10	2.03 ± 0.48	111.20 ± 13.20	29.50 ± 3.47	74.50 ± 11.22	35.33 ± 4.55	5.17 ± 0.17	1.00 ± .00

Values are mean ± S.E.M. (n = 8). *p* > 0.05 vs. distilled water (One-way ANOVA followed by Dunnett’s posttest).

### Effect of DAS-77 on serum electrolyte

There was a significant decrease (*p* < 0.05, 0.001) in the concentration of K^+^ in the groups treated with the formulation at the doses of 80 mg/kg (5.98 ± 0.25 mmol/L), 400 mg/kg (5.04 ± 0.10 mmol/L), and 2000 mg/kg (5.19 ± 0.14 mmol/L) when compared with the group treated with distilled water (7.52 ± 0.47 mmol/L) (Table 9). There was also a significant increase (*p* < 0.05) in the concentration of Ca^2+^ in the group treated with DAS-77 at the dose of 2000 mg/kg (1.25 ± 0.04 mg/dL) when compared with the distilled water treated group (1.09 ± 0.05 mg/dL). These effects were reversed after 30 days of cessation of administration of DAS-77 (Table 10).

**Table 9:** Effect of DAS-77 on serum electrolytes in the main study

**Treatment**	**Dose (mg/kg)**	**Main study**				
		**Na**^**+**^**(mmol/L)**	**K**^**+**^**(mmol/L)**	**Cl**^**−**^**(mmol/L)**	**HCO**_**3**_^**−**^**(mmol/L)**	**Ca**^**2+**^**(mg/dl)**
**Distilled water**	(10 ml/kg)	156.60 ± 4.44	7.52 ± 0.47	100.90 ± 3.73	10.56 ± 1.45	1.09 ± 0.05
**DAS-77**	80	150.30 ± 0.59	5.98 ± 0.25^*^	105.40 ± 0.38	10.13 ± 0.58	1.05 ± 0.03
**DAS-77**	400	152.70 ± 3.39	5.04 ± 0.10^***^	105.10 ± 0.53	12.70 ± 0.50	1.20 ± 0.03
**DAS-77**	2000	149.70 ± 0.90	5.19 ± 0.14^***^	104.70 ± 0.90	13.20 ± 0.84	1.25 ± 0.04^*^

Values are mean ± S.E.M. (n = 10). ^*^p < 0.05, ^***^p < 0.001 vs. distilled water (One-way ANOVA followed by Dunnett’s posttest).

**Table 10:** Effect of DAS-77 on serum electrolytes in the reversibility study

**Treatment**	**Dose (mg/kg)**	**Reversibility study**				
		**Na**^**+**^ **(mmol/L)**	**K**^**+**^**(mmol/L)**	**Cl**^**−**^ **(mmol/L)**	**HCO**_**3**_^**−**^**(mmol/L)**	**Ca**^**2+**^**(mg/dl)**
**Distilled water**	(10 ml/kg)	154.90 ± 1.29	5.19 ± 0.17	104.50 ± 0.93	11.38 ± 0.68	1.18 ± 0.05
**DAS-77**	80	154.80 ± 0.64	4.99 ± 0.17	106.70 ± 0.71	12.33 ± 0.88	1.12 ± 0.05
**DAS-77**	400	153.30 ± 0.80	5.09 ± 0.15	105.80 ± 1.07	11.63 ± 0.60	1.15 ± 0.04
**DAS-77**	2000	158.00 ± 1.07	5.10 ± 0.15	108.20 ± 1.17	11.83 ± 0.60	1.08 ± 0.05

Values are mean ± S.E.M. (n = 10). *p* > 0.05 vs. distilled water (One-way ANOVA followed by Dunnett’s posttest).

### Effect of DAS-77 on antioxidant enzymes in rat liver, kidney, brain and testes

There was no significant difference in the levels of the various antioxidant enzymes and MDA in the liver (Table 11) and brain (Table 12) but in the kidney, there was a significant increase (*p* < 0.05, 0.01) in the level of catalase in the groups treated with the formulation at doses of 80 mg/kg (58.21 ± 13.80 u/mg) and 2000 mg/kg (44.41 ± 4.75 u/mg) compared with the distilled water treated group (17.66 ± 5.22 u/mg) (Table 13). There was also a significant decrease (*p* < 0.05, 0.01) in the level of MDA in the groups treated with DAS-77 at doses of 80 mg/kg (0.11 ± 0.03 nm/mg), 400 mg/kg (0.07 ± 0.02 nm/mg), and 2000 mg/kg (0.13 ± 0.03 nm/mg) compared with the group treated with distilled water (0.27 ± 0.08 nm/mg). The effects on catalase and MDA in the kidney were reversed upon discontinuation of treatment for 30 days (Table 14).

**Table 11:** Effect of DAS-77 on antioxidant enzymes in rat liver in the main study

**Treatment**	**Dose (mg/kg)**	**Main study**					
		**GSH (nm/mg)**	**SOD (u/mg)**	**CAT** **(u/mg)**	**Peroxidase (u/mg)**	**MDA (nm/mg)**	**Protein** **(mg)**
**Distilled water**	(10 ml/kg)	1.78 ± 0.40	4.25 ± 1.31	19.62 ± 6.04	1.27 ± 0.39	0.11 ± 0.05	52.68 ± 6.14
**DAS-77**	80	1.75 ± 0.17	6.65 ± 0.39	30.65 ± 1.81	2.08 ± 0.12	0.04 ± 0.00	61.64 ± 6.92
**DAS-77**	400	2.07 ± 0.15	5.59 ± 1.03	25.76 ± 4.74	1.75 ± 0.32	0.11 ± 0.03	43.80 ± 5.34
**DAS-77**	2000	2.03 ± 0.26	6.62 ± 1.05	30.49 ± 4.81	2.08 ± 0.33	0.11 ± 0.02	47.08 ± 4.04

Values are mean ± S.E.M. (n = 5). *p* > 0.05 vs. distilled water (One-way ANOVA followed by Dunnett’s posttest).

**Table 12:** Effect of DAS-77 on antioxidant enzymes in rat brain in the main study

**Treatment**	**Dose (mg/kg)**	**Main study**					
		**GSH (nm/mg)**	**SOD (u/mg)**	**CAT (u/mg)**	**Peroxidase (u/mg)**	**MDA (nm/mg)**	**Protein (mg)**
**Distilled water**	(10 ml/kg)	1.35 ± 0.29	3.94 ± 1.26	18.19 ± 5.80	1.24 ± 0.39	0.23 ± 0.02	30.68 ± 3.80
**DAS-77**	80	1.89 ± 0.32	3.13 ± 0.75	14.44 ± 3.44	0.98 ± 0.23	0.28 ± 0.11	38.27 ± 6.34
**DAS-77**	400	1.68 ± 0.28	1.94 ± 0.28	8.96 ± 1.30	0.61 ± 0.09	0.18 ± 0.01	29.65 ± 4.47
**DAS-77**	2000	1.83 ± 0.30	2.38 ± 0.52	10.93 ± 2.42	0.74 ± 0.17	0.21 ± 0.02	27.47 ± 3.10

**Table 13:** Effect of DAS-77 on antioxidant enzymes in rat kidney in the main study

**Treatment**	**Dose (mg/kg)**	**Main study**					
		**GSH (nm/mg)**	**SOD (u/mg)**	**CAT** **(u/mg)**	**Peroxidase** **(u/mg)**	**MDA (nm/mg)**	**Protein (mg)**
**Distilled water**	(10 ml/kg)	1.69 ± 0.15	3.41 ± 0.73	17.66 ± 5.22	1.26 ± 0.34	0.27 ± 0.08	28.94 ± 5.64
**DAS-77**	80	2.76 ± 0.83	12.31 ± 3.01	58.21 ± 13.80^**^	3.96 ± 0.94	0.11 ± 0.03^*^	50.22 ± 10.34
**DAS-77**	400	2.12 ± 0.21	7.69 ± 0.53	35.46 ± 2.43	2.32 ± 0.15	0.07 ± 0.02^**^	51.13 ± 3.57
**DAS-77**	2000	2.17 ± 0.18	9.63 ± 1.03	44.41 ± 4.75^*^	3.02 ± 0.32	0.13 ± 0.03^*^	35.22 ± 2.57

Values are mean ± S.E.M. (n = 5). ^*^p < 0.05, ^**^p < 0.01 vs. distilled water (One-way ANOVA followed by Dunnett’s posttest).

**Table 14:** Effect of DAS-77 on antioxidant enzymes in rat kidney in the reversibility study

**Treatment**	**Dose (mg/kg)**	**Reversibility study**					
		**GSH (nm/mg)**	**SOD (u/mg)**	**CAT (u/mg)**	**Peroxidase (u/mg)**	**MDA (nm/mg)**	**Protein (mg)**
**Distilled water**	(10 ml/kg)	2.03 ± 0.37	3.30 ± 0.45	15.24 ± 2.08	1.04 ± 0.14	0.13 ± 0.01	32.56 ± 1.70
**DAS-77**	80	2.22 ± 0.46	3.48 ± 0.59	16.07 ± 2.70	1.09 ± 0.18	0.15 ± 0.02	30.88 ± 4.35
**DAS-77**	400	1.09 ± 0.14	2.04 ± 0.18	9.43 ± 0.85	0.64 ± 0.06	0.15 ± 0.02	35.49 ± 4.60
**DAS-77**	2000	2.31 ± 0.42	3.05 ± 0.49	14.07 ± 2.24	0.96 ± 0.15	0.14 ± 0.02	34.32 ± 5.29

Values are mean ± S.E.M. (n = 5, main study). *p* > 0.05 vs. distilled water (One-way ANOVA followed by Dunnett’s posttest).

There were significant differences in the level of antioxidant enzymes in the liver across the treatment groups after a 30 day cessation of therapy (Table 15). At the dose of 80 mg/kg, there were significant increases (*p* < 0.05, 0.01, 0.001) in the levels of GSH (3.69 ± 0.55 vs. 1.45 ± 0.22 nm/mg in control), SOD (5.82 ± 0.72 vs. 3.24 ± 0.33 u/mg in control), catalase (26.87 ± 3.33 vs. 14.94 ± 1.54 u/mg in control), and peroxidase (1.83 ± 0.23 vs. 0.10 ± 0.10 u/mg in control).

**Table 15:** Effect of DAS-77 on antioxidant enzymes in rat liver in the reversibility study

**Treatment**	**Dose (mg/kg)**	**Reversibility study**					
		**GSH (nm/mg)**	**SOD (u/mg)**	**CAT** **(u/mg)**	**Peroxidase (u/mg)**	**MDA (nm/mg)**	**Protein** **(mg)**
**Distilled water**	(10 ml/kg)	1.45 ± 0.22	3.24 ± 0.33	14.94 ± 1.54	0.10 ± 0.10	0.19 ± 0.02	34.68 ± 1.91
**DAS-77**	80	3.69 ± 0.55^***^	5.82 ± 0.72^**^	26.87 ± 3.33^**^	1.83 ± 0.23^**^	0.19 ± 0.02	24.41 ± 2.52^**^
**DAS-77**	400	1.70 ± 0.31	3.62 ± 0.56	16.72 ± 2.58	1.14 ± 0.18	0.22 ± 0.01	24.35 ± 1.40^*^
**DAS-77**	2000	2.29 ± 0.15	3.71 ± 0.14	17.10 ± 0.64	1.17 ± 0.04	0.93 ± 0.81	33.10 ± 3.66

Values are mean ± S.E.M. (n = 4). ^*^p < 0.05, ^**^p < 0.01, ^***^p < 0.001 vs. distilled water (One-way ANOVA followed by Dunnett’s posttest).

There was no significant difference in the levels of the various antioxidant enzymes and MDA in the brain in the reversibility study (Table 16).

**Table 16:** Effect of DAS-77 on antioxidant enzymes in rat brain in the reversibility study

**Treatment**	**Dose (mg/kg)**	**Reversibility study**					
		**GSH (nm/mg)**	**SOD (u/mg)**	**CAT (u/mg)**	**Peroxidase (u/mg)**	**MDA (nm/mg)**	**Protein (mg)**
**Distilled water**	(10 ml/kg)	3.67 ± 0.70	5.36 ± 0.91	24.72 ± 4.20	1.68 ± 0.29	0.25 ± 0.04	16.76 ± 0.94
**DAS-77**	80	2.49 ± 0.41	4.36 ± 0.40	20.12 ± 1.86	1.37 ± 0.13	0.28 ± 0.04	16.69 ± 1.20
**DAS-77**	400	2.15 ± 0.27	3.53 ± 0.33	16.29 ± 1.55	1.11 ± 0.11	0.33 ± 0.04	20.28 ± 1.22
**DAS-77**	2000	3.35 ± 1.20	5.26 ± 1.62	24.26 ± 7.49	1.65 ± 0.51	0.27 ± 0.04	20.76 ± 2.90

Values are mean ± S.E.M. (n = 4). *p* > 0.05 vs. distilled water (One-way ANOVA followed by Dunnett’s posttest).

In respect of the testes and DAS-77 treatment dose of 80 mg/kg, there were significant increases (*p* < 0.01) in the levels of GSH (3.58 ± 0.75 nm/mg), SOD (5.75 ± 0.82 u/mg), catalase (26.55 ± 3.79 u/mg), and peroxidase (1.80 ± 0.26 u/mg) compared with the distilled water treated groups (1.12 ± 0.16 nm/mg, 2.80 ± 0.32 u/mg, 13.48 ± 1.58 u/mg, and 0.95 ± 0.12 u/mg control values, respectively) (Table 17).

**Table 17:** Effect of DAS-77 on antioxidant enzymes in rat testes

		**GSH (nm/mg)**	**SOD (u/mg)**	**CAT** **(u/mg)**	**Peroxidase (u/mg)**	**MDA (nm/mg)**	**Protein (mg)**
**Distilled water**	(10 ml/kg)	1.12 ± 0.16	2.80 ± 0.32	13.48 ± 1.58	0.95 ± 0.12	0.26 ± 0.02	25.48 ± 1.87
**DAS-77**	80	3.58 ± 0.75^**^	5.75 ± 0.82^**^	26.55 ± 3.79^**^	1.80 ± 0.26^**^	0.28 ± 0.06	24.19 ± 2.22
**DAS-77**	400	0.65 ± 0.11	1.86 ± 0.23	8.57 ± 1.05	0.59 ± 0.07	0.19 ± 0.01	35.08 ± 2.95
**DAS-77**	2000	1.46 ± 0.47	2.44 ± 0.18	11.26 ± 0.85	0.77 ± 0.06	0.19 ± 0.06	38.65 ± 5.58^*^

Values are mean ± S.E.M. (n = 4). ^*^p < 0.05, ^**^p < 0.01 vs. distilled water (One-way ANOVA followed by Dunnett’s posttest).

### Effect of DAS-77 on histopathological presentations

A summary of the histopathological presentations in the various organs is shown in Table 18.

**Table 18:** Histopathological presentations

**Organs**	**Distilled water** **(10 ml/kg)**	**DAS-77** **(80 mg/kg)**	**DAS-77** **(400 mg/kg)**	**DAS-77** **(2000 mg/kg)**
Liver	Normal	Normal	Severe congestion	Congestion
Kidney	Normal	Normal	Normal	Normal
Heart	Normal	Normal	Normal	Normal
Lungs	Normal	Normal	Normal	Normal
Brain	Normal	Normal	Cerebral oedema	Normal
Pancreas	Normal	Normal	Normal	Normal
Spleen	Congestion	Congestion	Congestion	Congestion
Ovaries	Normal	Normal	Normal	Normal
Testes	Normal	Normal	Normal	Normal

#### Liver

**Figure 5:**
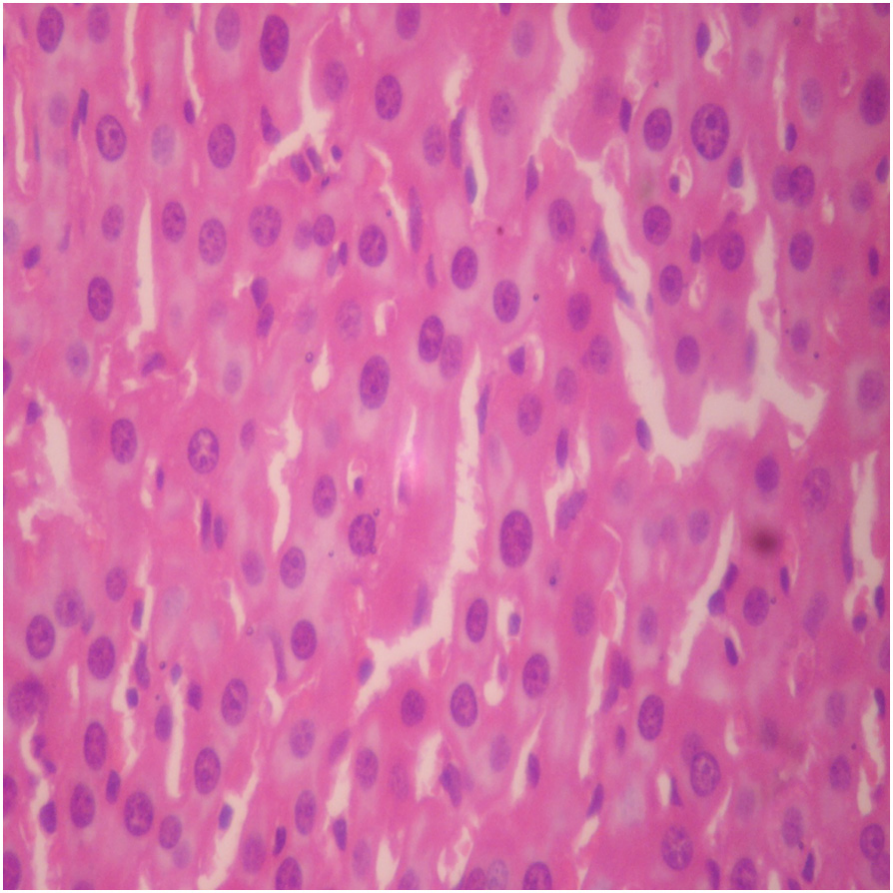
Distilled water group showing the liver (normal) (× 400).

**Figure 6:**
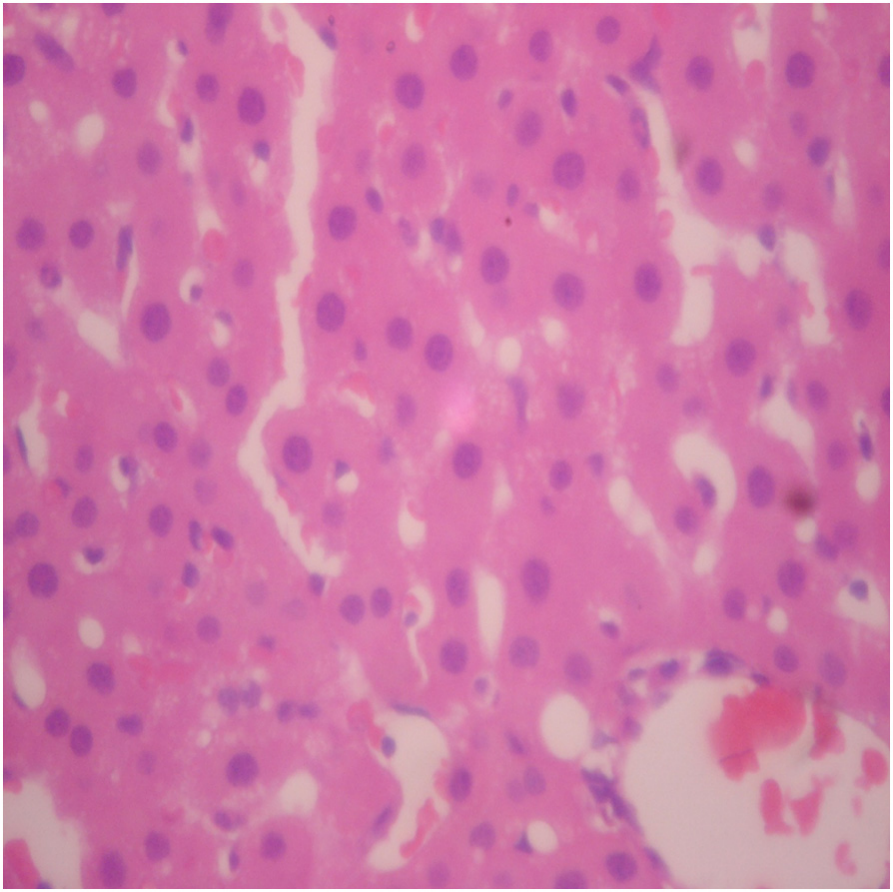
DAS-77 (80 mg/kg) group showing the Liver (normal) (× 400).

**Figure 7:**
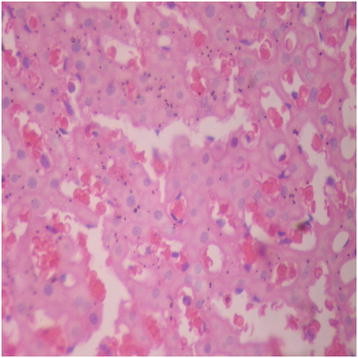
DAS-77 (400 mg/kg) group showing the liver (congested) (× 400).

**Figure 8:**
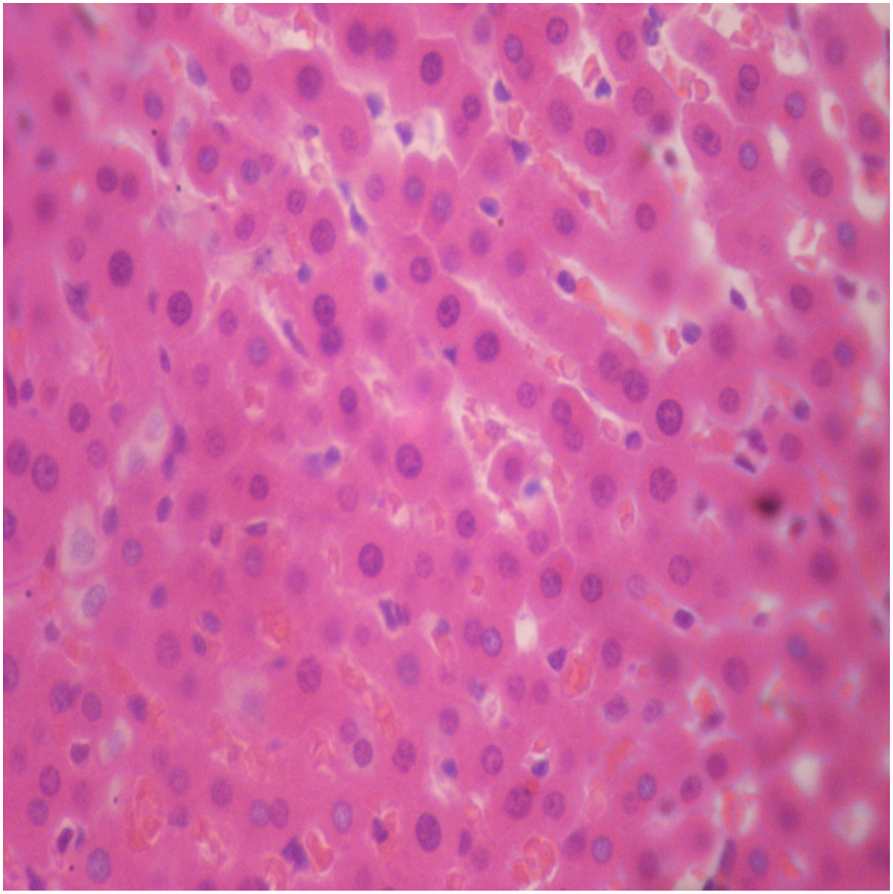
DAS-77 (2000 mg/kg) group showing the liver (congested) (× 400).

There were no adverse histopathological presentations observed in the distilled water and DAS-77 (80 mg/kg) treatment groups. The liver appeared normal with preserved hepatic architecture, hepatocytes arranged as radial plates, and having eosinophilic cytoplasm and central nuclei. No cytoplasmic inclusions were seen and no portal inflammation (Figures 5 and 6). Congestion was observed in the liver at doses of 400 and 2000 mg/kg with the sinusoidal spaces showing vascular congestion (Figures 7 and 8).

#### Kidney

There were no adverse histopathological presentations observed in all the treatment groups. Normocellular glomerular tufts were displayed on a background containing tubules. No necrosis was observed.

#### Brain

**Figure 9:**
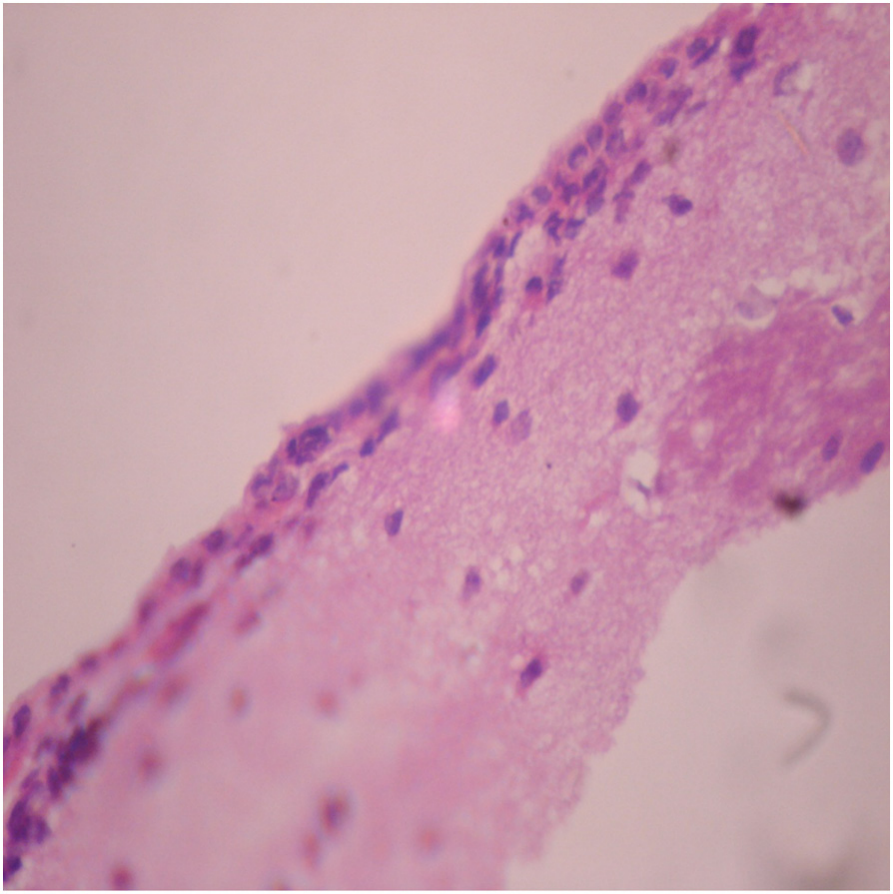
Distilled water group showing the brain (normal) (× 400).

**Figure 10:**
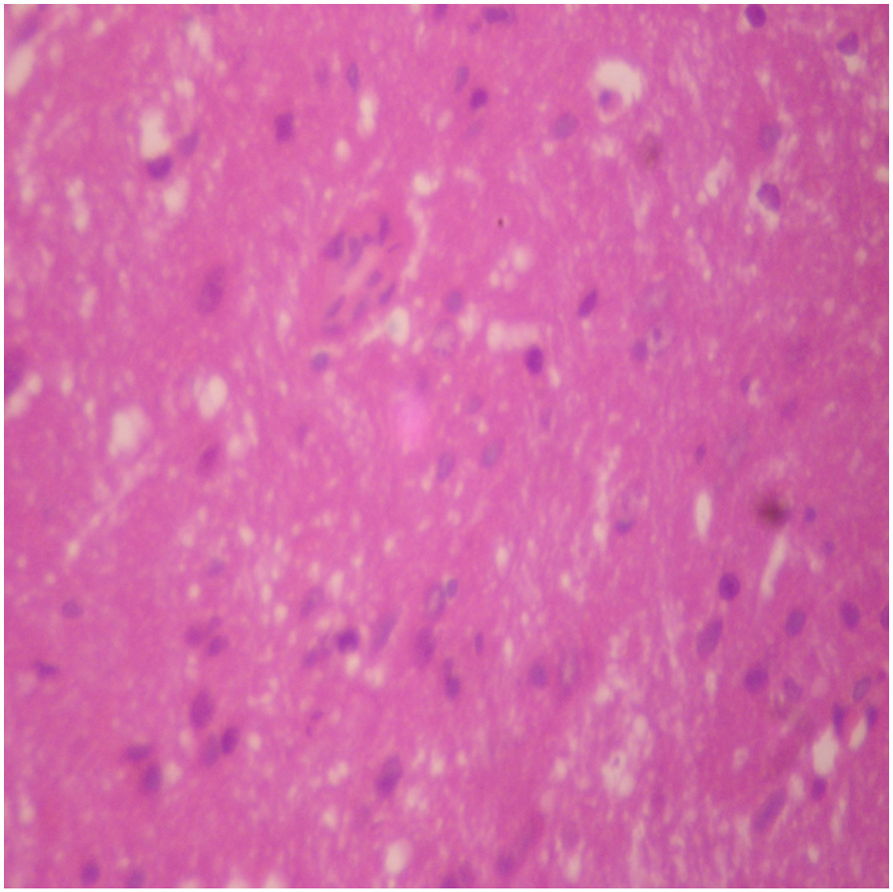
DAS-77 (80 mg/kg) group showing the brain (normal) (× 400).

**Figure 11:**
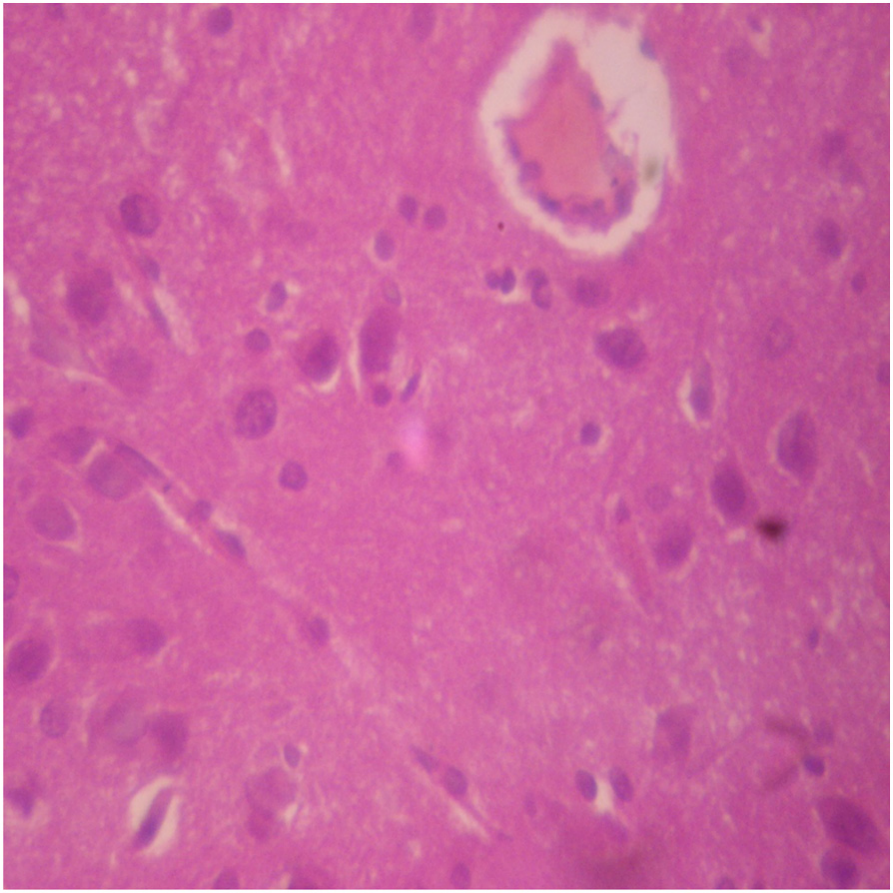
DAS-77 (400 mg/kg) group showing the brain (cerebral oedema).

**Figure 12:**
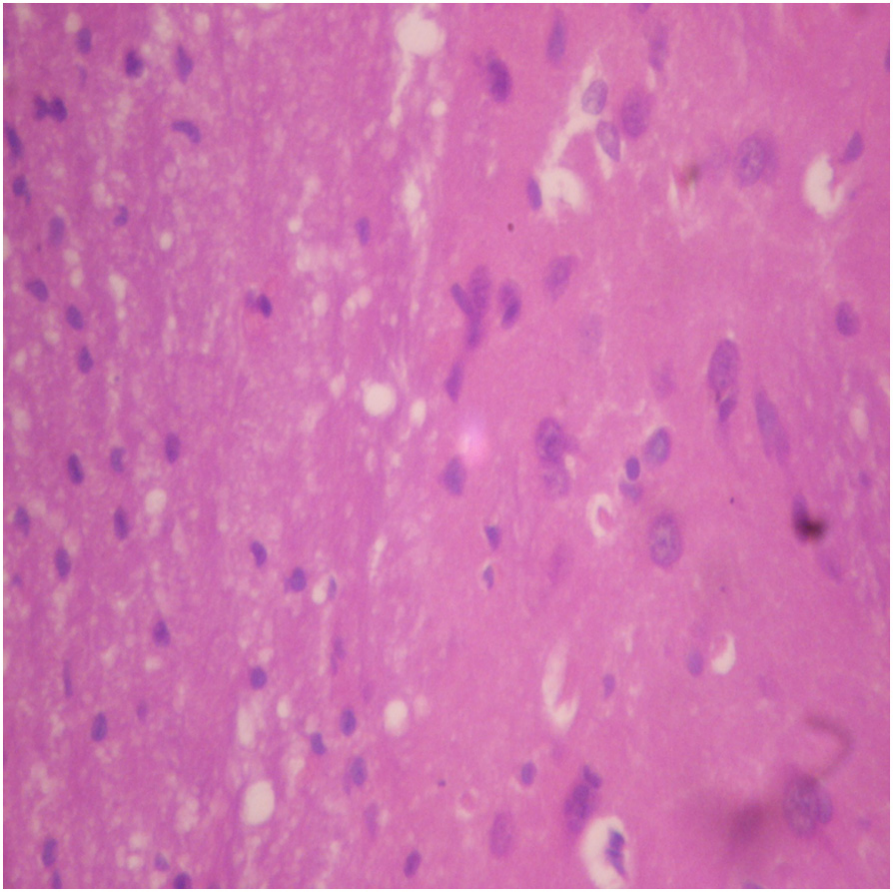
DAS-77 (2000 mg/kg) group showing the brain (normal) (× 400).

Generally, there were no adverse histopathological presentations observed in the treatment groups but cerebral oedema was observed in the 400 mg/kg group (Figures 9, 10, 11 and 12). Neuron cell bodies were displayed on a loose fibrillary background. Prominent perineuronal and perivascular halos or clearing (Virchow Robbins spaces) were seen.

#### Lungs

The lungs were normal in all the treatment groups. The alveolar air spaces were surrounded by interstitium containing few blood vessels and inflammatory cells.

#### Heart

The heart was normal in all the treatment groups. The cardiac myocytes were arranged in interlacing and parallel array. Their nuclei were spindle shaped and elongated.

#### Spleen

**Figure 13:**
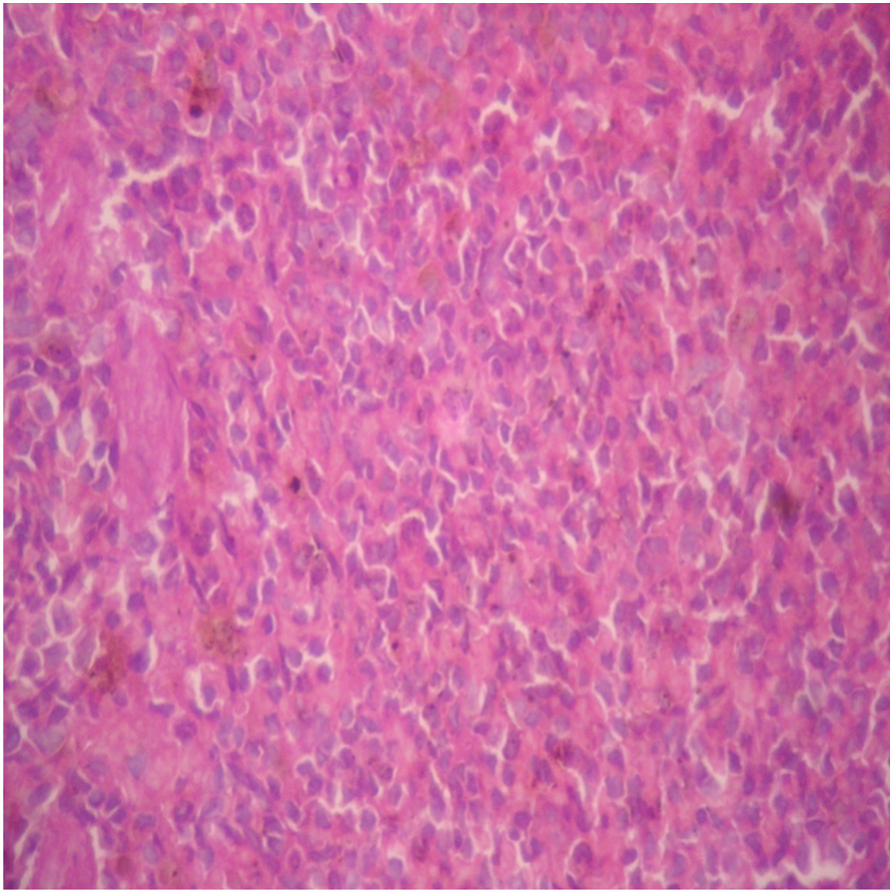
Distilled water group showing the spleen (normal) (× 400).

**Figure 14:**
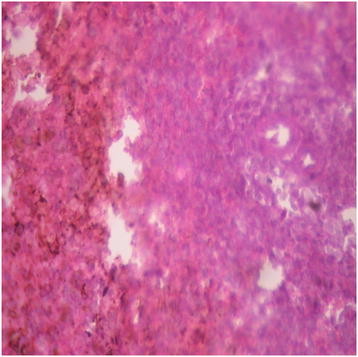
DAS-77 (80 mg/kg) group showing the spleen (congested) (× 400).

**Figure 15:**
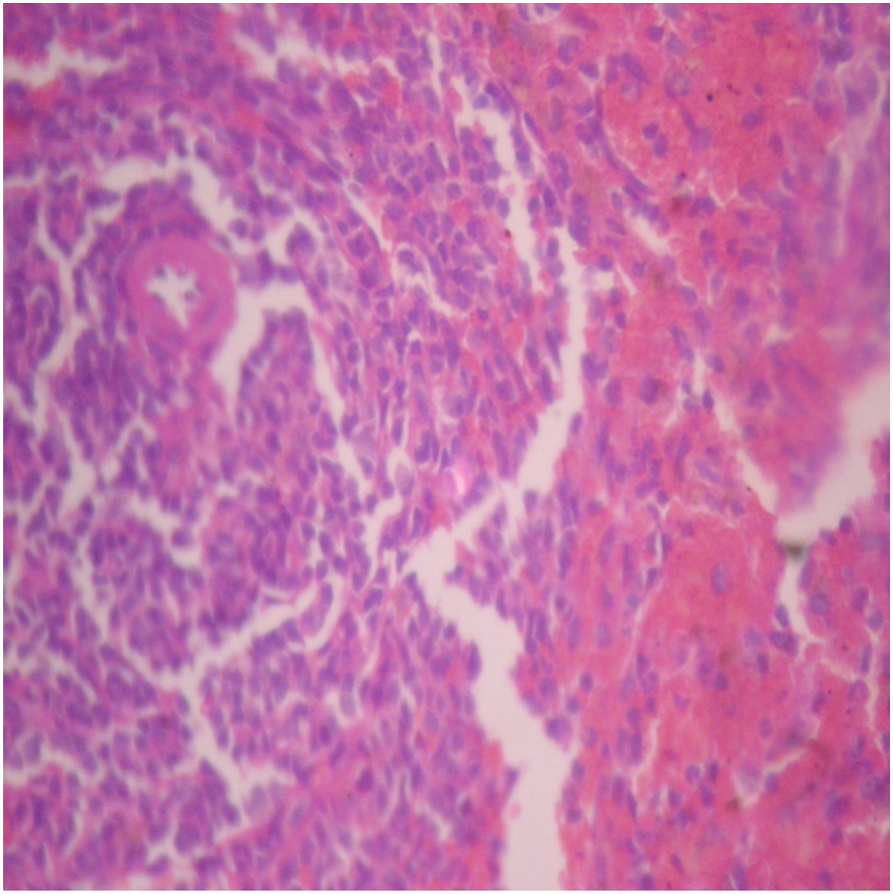
DAS-77 (400 mg/kg) group showing the spleen (congested) (× 400).

**Figure 16:**
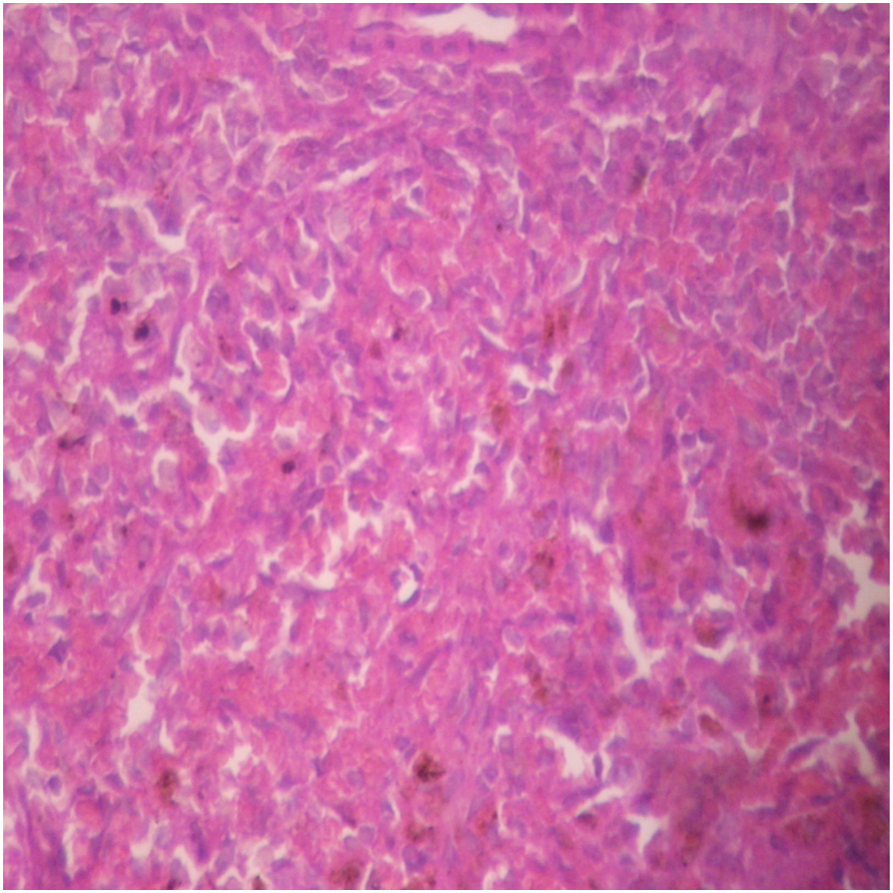
DAS-77 (2000 mg/kg) group showing the spleen (congested) (× 400).

Presentations in the distilled water group were normal. The spleen was congested in all the DAS-77 treatment groups with the background splenic stroma showing engorgement of sinusoids by red blood cells (Figures 13, 14, 15 and 16).

#### Pancreas

The pancreas was normal in all the treatment groups. Closely packed acini, separated by delicate fibrocollagenous stroma that transmits blood vessels were observed.

#### Testes

Normal histopathological presentations were observed in all the treatment groups. Histologic section shows seminiferous tubules lined by germ cells in various stages of development (the spermatogenic series), and containing luminal spermatozoa. Leydig cells were seen in the interstitium.

#### Ovaries

Normal histopathological presentations were observed in all the treatment groups. Histologic section of ovary shows follicles at various stages of development as well as corpora lutea, displayed on a fibrocellular stroma.

#### Mortality

Mortality recorded in the distilled water and DAS-77 (80, 400 and 2000 mg/kg) treatment groups within 30, 60 and 90 days, and the reversibility study period were 0/20, 1/20, 1/20 and 0/20 (10%); 0/20, 1/20, 0/20 and 0/20 (5%); 1/20, 1/20, 0/20 and 0/20 (10%); and 1/20, 2/20, 0/20 and 1/20 (20%), respectively.

## Discussion

Worldwide, various medicinal plants and botanical drugs have been widely adapted as primary therapeutic agents or supplements for treating various human ailments [33]. Based on the findings that herbal medicines are abused, there is a great need to look into their acute and chronic toxicity effects.

DAS-77 did not induce lethality in mice when administered orally up to 20 g/kg in divided doses. Hence, the herbal preparation can be said to be safe when administered orally according to the assertion of Clarke and Clarke [34] that a substance that does not produce lethality up to 10 g/kg orally is relatively non-toxic. Visible signs of delayed toxicity were not observed. However, the LD_50_ when DAS-77 was administered intraperitoneally was estimated to be 1122.0 mg/kg. The behavioral changes observed during acute exposures through the intraperitoneal route were writhing, grooming, increased locomotor activity and convulsion.

Considering the therapeutic dose of DAS-77 (400 mg/kg), the hematological analysis showed an increase in neutrophils which possibly suggest a boost in defense, mainly acute response to infections or antigens. This is based on the fact that neutrophils are essential first line defense of the body against infections or introduction of antigens and neutropenia (decrease in neutrophils) makes an individual highly susceptible to infections [35]. Other hematological parameters were not affected. There was an increase in the weight of the ovaries and this could suggest preparation for ovulation cycle as the histopathological findings showed normal presentations in the ovaries [36]. Antioxidant enzymes in the liver and brain did not show any significant change. However, a decrease in the level of MDA was observed in the liver. Malondialdehyde is a non-lipophilic peroxidation product of polyunsaturated fatty acids containing three or more methylene interrupted double bonds. Production of MDA reflects lipid peroxidation caused by oxidative damage [37]. Food and water intake were reduced and this may be an indicator that DAS-77 has significant effect on the CNS based on the fact that some CNS stimulants cause anorexia [38]. The herbal formulation at the therapeutic dose did not affect sperm motility, count and morphology. Biochemical estimations showed an increase in HDL. Epidemiological studies have shown that high concentrations of HDL confer protective value against cardiovascular diseases such as ischemic stroke and myocardial infarction. Low concentrations of HDL increase the risk for atherosclerotic diseases [39]. This shows a potential for cardioprotective effect at this dose. There was a decrease in the level of potassium in the serum which suggests a potential for DAS-77 to cause electrolyte imbalance at this dose. Potassium is essential for many body functions, including muscle and nerve activity. The electrochemical gradient of potassium between the intracellular and extracellular space is essential for nerve function. In particular, potassium is needed to repolarize the cell membrane to a resting state after an action potential has passed. Decreased potassium levels in the extracellular space will cause hyperpolarization of the resting membrane potential. The reversibility studies showed that the effects induced by DAS-77 at the therapeutic dose in the main study were reversed. This suggests that these effects were not continuous after withdrawal of administration.

In respect of the subtherapeutic dose (80 mg/kg), DAS-77 had no significant effect on organ weights compared to control. There were no significant changes in food and water intake, and weight of animals. Hematological findings showed increase in the percentage of neutrophils which possibly suggests a boost in the immune response [35]. There was no effect on the motility, count and morphology of sperm cells. The testicular level of GSH, SOD, CAT and peroxidase were increased with no significant effect on MDA relative to control. The level of MDA and antioxidant enzymes in the liver and brain were not affected. However, a decrease in the level of MDA was observed in the kidneys. The level of MDA is a signal of lipid peroxidation and oxidative damage and the low level observed in the kidney shows a reduced possibility of oxidative stress [37]. Serum electrolyte estimation showed a decrease in potassium levels which suggest electrolyte imbalance. This would have an effect on muscle contraction and activities in the nervous system. Biochemical assays showed an increase in HDL, indicating a potential for protective value against cardiovascular diseases such as ischemic stroke and myocardial infarction [39]. Hence, DAS-77 at the dose of 80 mg/kg may possibly elicit cardioprotective effect. The effects produced by the herbal preparation at this dose were reversed upon cessation of administration.

DAS-77 at the supratherapeutic dose (2000 mg/kg) also produced an increase in the size of the ovaries. This may be due to preparation for ovulation in view of the fact that histopathological examinations showed normal presentation without any inflammation. There was also a decrease in food intake which possibly suggests anorexia. This may be an indication of impact on the CNS at this dose. There was a decrease in the WBC count which could be that the formulation suppresses immunity at this dose. Differential WBC analysis revealed an increase in the percentage of neutrophils possibly suggesting a boost in the acute immune response of the body [35]. There was a tremendous effect on sperm motility, count and morphology. Motility and count were significantly reduced and the percentage of abnormal cells was high relative to control suggesting significant reduction in fertility. This shows that DAS-77 at this dose has a potential for use as a male contraceptive as all the components of a potent sperm cell were reversibly affected. Antioxidant enzymes and MDA estimation in the kidney showed an increase in catalase and decrease in MDA levels which means that there is enhanced conversion of hydrogen peroxide to water and gaseous oxygen. Hydrogen peroxide, a free radical, is a harmful by-product of many normal metabolic processes. To prevent damage, it must be quickly converted into other less dangerous substances. In view of this, catalase is frequently used by cells to rapidly catalyze the decomposition of hydrogen peroxide into less reactive gaseous oxygen and water molecules [40]. A reduced MDA level also shows ability to mop up dangerous species of free radicals [37]. DAS-77 at the supratherapeutic dose produced a decrease in the level of potassium. As stated earlier, reduction in potassium levels in the extracellular space will cause hyperpolarization of the resting membrane potential which may affect activities of the nervous system. There was a significant reduction in the levels of AST and ALT. The levels of these enzymes are raised in acute liver damage. AST is also present in red blood cells, cardiac muscle, skeletal muscle, kidney and brain tissue, and may be elevated due to damage to these sources as well. AST is defined as a biochemical marker for the diagnosis of acute myocardial infarction [41]. ALT is fairly specific being found largely in the liver [8] and it is commonly used as a biomarker for liver problems [42]. The reduction in the levels of AST and ALT may suggest potential for hepatoprotective action. An independent study is however necessary to ascertain this. The reversibility study showed that the effects produced by the herbal preparation at the supratherapeutic dose in the main study were reversed.

Histopathological presentations in this study give credence to the results obtained in the assessment of hematological and biochemical parameters with the 90 day administration of DAS-77. These findings may suggest that the mortality recorded in the course of this study may be administration related.

## Conclusion

Findings in the acute toxicity test suggest that DAS-77 is practically non-toxic when administered orally. The results obtained in respect of the chronic toxicity study suggest that DAS-77 is relatively safe when administered orally for an extended period at subtherapeutic and therapeutic doses. The phytomedicine at these doses showed potential for boosting components of the immune system and protecting the kidneys, liver and cardiovascular system. These findings provide a justification for specifically designed studies to further investigate these possible beneficial activities. However, DAS-77 at these doses showed a tendency to reversibly cause electrolyte imbalance. Observations in this study also revealed that the supratherapeutic dose of DAS-77 is endowed with the potential beneficial effects observed with the lower doses but with the risk of reversible electrolyte imbalance also and additionally, reversible sterility in males.

## Abbreviations

LD_50_: Median lethal dose; EDTA: Ethylenediamine-tetra Acetate; MDA: Malondialdehyde; PCV: Packed Cell Volume; RBC: Red Blood Cell; Hb: Hemoglobin; WBC: White Blood Cell; MCHC: Mean Cell Hemoglobin Concentration; MCV: Mean Red Cell Volume; MCH: Mean Cell Hemoglobin; HDL: High Density Lipoprotein; LDL: Low Density Lipoprotein; TG: Triglycerides; ALP: Alkaline Phosphatase; AST: Aspartate Transaminase; ALT: Alanine Transaminase; GSH: Reduced Glutathione; SOD: Superoxide Dismutase; CAT: Catalase; GPx: Glutathione Peroxidase.

## Competing interests

The authors declare that there are no conflicts of interest in respect of this study.

## Authors’ contributions

AJA and OA designed the study and assisted SOA in its conduct, CCA was responsible for the histopathological assessment, SOA and AJA drafted the manuscript, and OOA coordinated the study and revised the manuscript. All authors reviewed and interpreted the data, read and approved the final manuscript.

## Pre-publication history

The pre-publication history for this paper can be accessed here:

http://www.biomedcentral.com/1472-6882/12/79/prepub
